# Physiopathology of Bone Modifications in **β**-Thalassemia

**DOI:** 10.1155/2012/320737

**Published:** 2012-05-30

**Authors:** Carlo Perisano, Emanuele Marzetti, Maria Silvia Spinelli, Cinzia Anna Maria Callà, Calogero Graci, Giulio Maccauro

**Affiliations:** ^1^Department of Orthopaedics and Traumatology, University Hospital Agostino Gemelli, Catholic University of the Sacred Heart School of Medicine, Largo A. Gemelli 1, 00168 Rome, Italy; ^2^Department of Biochemistry and Clinical Biochemistry, University Hospital Agostino Gemelli, Catholic University of the Sacred Heart School of Medicine, Largo A. Gemelli 1, 00168 Rome, Italy

## Abstract

**β**-thalassemia major (**β**TM) or Cooley anemia is characterized by significantly reduced or absent synthesis of **β**-globin chains, which induces important pathologic consequences including hemolytic anemia, altered erythropoiesis, and bone marrow overstimulation. The pathogenesis of bone changes in patients with **β**TM is not yet completely understood. However, an unbalance in bone mineral turnover resulting from increased resorption and suppression of osteoblast activity has been detected in **β**TM patients. The abnormal regulation of bone metabolism may be related to hormonal and genetic factors, iron overload and iron chelation therapy, nutritional deficits, and decreased levels of physical activity. Here, we review the most recent findings on the physiopathology of bone abnormalities in **β**TM. Clinical presentation and radiological features of **β**TM-related bone changes are also discussed.

## 1. Introduction

*β*-thalassemia, firstly described by Cooley and Lee [1], comprises a group of inherited, autosomal, recessive, and hematologic disorders characterized by decreased or absent synthesis of *β*-globin chains. The mature hemoglobin (Hb) molecule is a tetramer composed of two *α*-globin and two *β*-globin chains, along with a heme prosthetic group. *β*-globin synthesis is controlled by one gene located on each chromosome 11 [2]. Defects are usually secondary to point mutations and rarely occur as a consequence of deletions [2]. In *β*-thalassemia, *β*-globin chain production can range from near to normal to completely absent, leading to varying degrees of excess *α*-globin chains and disease severity [2]. *β*-thalassemia trait (minor), resulting from heterozygosity for *β*-thalassemia, is clinically asymptomatic and manifests with microcytosis and mild anemia. *β*-thalassemia intermedia comprises a clinically and genotypically heterogeneous group of disorders, ranging in severity from the asymptomatic carrier state to severe, transfusion-dependent disease. *β*-thalassemia major (*β*TM) or Cooley anemia is characterized by severely reduced or absent synthesis of *β*-globin chains from both genes, with symptoms and signs beginning at about six months of age (abdominal swelling, growth retardation, irritability, jaundice, pallor, skeletal abnormalities, and splenomegaly) [2].

In *β*TM, the defective synthesis of *β* chains, together with excess *α* chains, leads to hemolytic anemia, altered erythropoiesis, reduced erythrocyte survival, and bone marrow overstimulation [1–4]. Patients need blood transfusions to correct anemia and iron-chelating therapy to control iron overload [1–4]. Anemia, excess body iron, and iron-chelation therapy can result in endocrine disorders (e.g., diabetes mellitus, hypogonadism, hypothyroidism, hypoparathyroidism, hypopituitarism, and Addison's disease), growth retardation, liver and cardiac failure, and splenomegaly. The latter can worsen anemia and occasionally causes thrombocytopenia and neutropenia, thereby increasing the risk of infections and hemostatic disorders [2–5]. Heart failure is the leading cause of death in patients with *β*TM [3, 6].

## 2. Epidemiology

The worldwide prevalence of *α*- and *β*-thalassemia trait is 1.7% [4]. Males and females are equally affected. The incidence of thalassemia trait is 4.4 per 10,000 live births [4]. *β*-thalassemia in its various presentations is more common in the Mediterranean area, Africa, and Southeastern Asia.

## 3. Pathogenesis of Bone Changes in *β*-Thalassemia

The pathogenesis of bone changes in *β*TM patients is not yet completely understood [7]. In spite of the improved treatment of the hematologic disorder and its complications, *β*-thalassemia patients exhibit an unbalance in bone mineral turnover with increased resorptive rates and suppression of osteoblast activity, resulting in diminished bone mineral density (BMD) more evident in the lumbar spine [8, 9]. Putative mechanisms involved in the pathogenesis of bone abnormalities in *β*TM are discussed in the following subsections.

### 3.1. Impairments in Osteoblast Activity

Mahachoklertwattana et al. [7, 10] reported growth retardation and delayed bone age, reduced BMD (especially of the lumbar spine), and low serum IGF-I levels in children and adolescents with *β*TM. In these patients, bone histomorphometry revealed increased osteoid thickness and delayed osteoid maturation and mineralization, indicating impaired bone matrix maturation and defective mineralization [7]. In addition, iron depots were detected along mineralization fronts and osteoid surfaces, while focal-thickened osteoid seams were found together with iron deposits. Dynamic bone formation studies revealed reduced bone formation rates. These findings indicate that delayed bone maturation and focal osteomalacia contribute to the pathogenesis of bone disease in suboptimally blood-transfused *β*TM patients with iron overload. Iron depots within bones and low circulating IGF-I levels may partly contribute to skeletal abnormalities [7].

Morabito et al. [11] showed that *β*TM patients displayed an unbalanced bone turnover, characterized by enhanced resorption rates (indicated by high levels of pyridinium cross-links) and a decreased neoformation phase (evidenced by low levels of osteocalcin, an osteoblast-derived protein) [11]. Voskaridou and colleagues [12] found increased serum levels of Dickkopf-1 (Dkk1), a soluble inhibitor of wingless type (Wnt) signaling, and sclerostin [13], a Wnt inhibitor, specifically expressed by osteocytes, in *β*TM patients. Higher circulating levels of Dkk1 and sclerostin correlated with reduced bone mineral density of lumbar spine and distal radius as well as with increased bone resorption and reduced bone formation markers. These findings indicate that disruption of Wnt signaling in patients with thalassemia and osteoporosis leads to osteoblast deregulation. Therefore, sclerostin and Dkk-1 have been proposed as potential targets for treatment in patients with thalassemia-induced osteoporosis [13].

### 3.2. Abnormal Osteoclast Activity

Besides impairments in osteoblast activity, which are thought to be a major cause of osteopenia/osteoporosis in *β*TM, an enhanced activation of osteoclasts is also invoked as a contributing factor [14]. This provides the rationale for the use of bisphosphonates, which are potent inhibitors of osteoclast function, for the management of *β*TM-induced osteoporosis [15].

An association between increased circulating levels of proresorptive cytokines and altered bone turnover has been detected in *β*TM patients [16]. The receptor activator of nuclear factor-kappa B (RANK)/RANK ligand (RANKL)/osteoprotegerin (OPG) pathway has recently been recognized as the final, dominant mediator of osteoclast proliferation and activation [9]. The OPG/RANKL system acts as an important paracrine mediator of bone metabolism also in thalassemic patients. Indeed, these patients showed no differences in plasma levels of OPG in the presence of higher circulating levels of RANKL, with consequent lower OPG/RANKL ratio and increased osteoclastic activity [16]. Urinary levels of pyridinium cross-links, a marker of bone resorption, were higher in *β*TM patients than controls and were positively correlated with plasma levels of RANKL, pointing to a central role of the OPG/RANKL system in development of bone abnormalities in *β*TM. It is suggested that the OPG/RANKL pathway may be involved in mediating the skeletal actions of sex steroids in *β*TM patients, as indicated by the negative correlation existing between serum levels of RANKL and sex hormones [16]. Therefore, the improvement of patient compliance to hormonal replacement therapy could correct the alterations of the OPG/RANKL system, potentially normalizing bone turnover. Furthermore, since the degree of bone resorption depends on Hb levels and the severity of hypogonadism, adequate hormonal replacement therapy and annual monitoring of bone conditions may be of benefit in young adult *β*TM patients [17].

The negative relationship between Hb and RANKL levels as well as between erythropoietin and OPG/RANKL ratio also suggests that medullary expansion may act through enhanced RANKL levels in increasing bone resorption. Indeed, anemia, by continuously stimulating erythropoietin synthesis and hence determining bone marrow hyperplasia, may increase bone resorption through enhanced RNAKL levels [11]. In addition, the expansion of bone marrow can cause mechanical interruption of bone, cortical thinning, bone distortion, and increased fragility [14].

### 3.3. Hormonal Factors

Hormonal abnormalities, including diabetes, thyroid/parathyroid dysfunction, and hypogonadism, are believed to underlie the altered bone turnover observed in *β*TM [14]. In female *β*TM patients, low estrogen and progesterone levels enhance osteoclast activity and reduce bone formation, while in males, low testosterone levels result in a decrease in its stimulatory effects on osteoblast proliferation and differentiation [14]. In addition, insufficiency of the GH-IGF-1 axis leads to impaired osteoblast proliferation and bone matrix formation, while increasing osteoclast activation [14].

### 3.4. Genetic Factors

Genetic factors have been shown to play a role in the pathogenesis of osteopenia/osteoporosis in *β*TM patients [14]. For instance, a polymorphism G→T or TT in the regulatory region of COLIA1 at the recognition site for transcription factor Sp1 is associated with the presence of osteoporosis [18]. Sp1 polymorphism occurs more frequently in females but is not specific to any ethnic group. In *β*TM male patients, the presence of the Sp1 mutation is associated with more severe osteoporosis of the spine and the hip compared with female patients [18]. In addition, male *β*TM patients who are heterozygous or homozygous at the polymorphic Sp1 site have lower BMD than females and no improvements in spinal osteoporosis in response to treatment with bisphosphonates [18]. Another study showed a consistent association between Sp1 polymorphism and vertebral osteoporosis in a sample of Italian *β*TM patients, suggesting the possibility that genotyping of the Sp1 site could be of clinical value for the identification of thalassemic patients at risk for osteoporosis and fractures [19].

Vitamin D receptor (VDR) polymorphisms at exon 2 (FokI) and intron 8 (BsmI) may be involved in determining the stature and BMD at femoral neck (FBMD) and lumbar spine (LBMD) in *β*TM patients [20]. Indeed, significantly shorter stature and lower LBMD and FBMD were observed in patients harboring the CC VDR genotype, while significant shorter height and lower LBMD have been reported in prepubertal and pubertal female patients with the BB VDR genotype [20].

### 3.5. Iron Overload and Iron-Chelation Therapy

Iron overload impairs osteoid maturation and inhibits local mineralization, resulting in focal osteomalacia [14]. In addition, the incorporation of iron in calcium hydroxyapatite affects the growth of crystals, leading to defective mineralization [14].

Deferoxamine, the most commonly used iron chelator, inhibits DNA synthesis, osteoblast and fibroblast proliferation, osteoblast precursor differentiation, and collagen formation, while enhancing osteoblast apoptosis [14].

### 3.6. Miscellanea

Nutritional deficits are commonly observed in *β*TM patients and may contribute to bone abnormalities. In particular, vitamin C deficiency can lead to impaired osteoblast activation and reduced collagen synthesis. Low vitamin D levels are associated with alterations in calcium/phosphate homeostasis, reduced osteoblast activity, and increased bone resorption rates [14]. Finally, decreased levels of physical activity, due to disease complications and/or overprotection, negatively influence bone turnover, leading to reduced bone formation and enhanced resorption [14].

## 4. Clinical Features

Bone marrow expansion and extramedullary hemopoiesis can result in the classical enlargement of cranial and facial bones with mongoloid appearance, as originally described by Cooley [1, 3]. Novel transfusion regimens and early iron-chelating therapy have improved the survival of *β*TM patients [21] and have substituted the marked bone abnormalities previously described [1] with less severe skeletal lesions. Yet, sequelae of osteopenia and severe osteoporosis represent the leading cause of morbidity in *β*TM patients [14, 22]. Indeed, the prevalence of osteoporosis in these patients is as high as 50%, with higher rates in males [23, 24].

In *β*TM patients, bone fractures range incidence between 38 and 41% and occur as a consequence of falls in over 50% of cases [14, 25]. Fractures more frequently involve the upper limb, while spine, hips, and pelvis are affected in 10% of cases [14, 25]. Due to the high bone fragility of *β*TM patients, fractures of long bones, especially those involving the femur, should be treated as pathological fractures and require the stabilization of the entire bone with intramedullary nailing [26].

*β*TM patients may also develop the so-called thalassemic osteoarthropathy, a nonerosive seronegative osteoarthropathy of varying severity, characterized by soft tissue swelling and pain, usually localized at the ankle joints [27]. Other skeletal abnormalities relatively common in *β*TM patients include lower and upper limb length discrepancy due to premature fusion of the epiphyseal line [28], axial deviation of the limbs, osteochondrosis, and short stature [14, 29, 30]. Involvement of the spine is frequent and can manifest as spinal deformities (e.g., scoliosis, kyphosis), vertebral collapse, cord compression, or intervertebral disc degeneration [9, 31–35].

## 5. Radiological Features

In *β*TM patients, the most evident radiological changes are those caused by intense marrow hyperplasia [36]. Such abnormalities include bone cortex thinning and widening of intratrabecular spaces, usually seen in hands, but also in the pelvis and ribs [36]. Extramedullary hemopoietic tissue sometimes grows beneath the periosteum, producing a scalloped cortex edge in hands, feet, tibiae, fibulae, knees, radii, and ulnae. In other cases, extramedullary hemopoietic tissues can appear as large intrathoracic masses, simulating paravertebral tumors. In the skull, significant thickening of the cranium can take place, and overgrowth of the facial bones can impede pneumatization of sinuses [36].

## 6. Conclusions

 Bone changes are frequent in *β*TM patients and occur as a consequence of the hematological disorder and its complications as well as iron overload, iron-chelation therapy, nutritional deficits, and sedentarism. The sequalae of osteoporosis, especially vertebral and long bone fractures, represent a major cause of morbidity in these patients. A better understanding of the pathogenetic mechanisms underlying bone abnormalities in *β*TM is needed to develop targeted treatments. As of now, the early detection of osteoporosis and the eventual institution of bisphosphonate treatment are the most effective strategies to reduce the incidence and severity of skeletal complications. The use of new-generation iron chelators may avoid the negative effects of deferoxamine on bone metabolism. Finally, the identification and correction of nutritional and hormonal deficits and the engagement in physical training programs should be pursued in *β*TM patients to reduce the incidence of osteoporosis and increase overall bone strength.
